# In vitro antibiotic susceptibility testing of *Brucella* isolates from Egypt between 1999 and 2007 and evidence of probable rifampin resistance

**DOI:** 10.1186/1476-0711-11-24

**Published:** 2012-08-28

**Authors:** Mohamed Abdel-Maksoud, Brent House, Momtaz Wasfy, Bassem Abdel-Rahman, Guillermo Pimentel, Gehan Roushdy, Erica Dueger

**Affiliations:** 1 U.S. Naval Medical Research Unit No. 3, PSC 452, 5000 Cairo, Egypt; 2 Central Public Health Laboratory, Cairo, Egypt; 3 U.S. Centers for Disease Control and Prevention, Atlanta, GA, USA

**Keywords:** *Brucella*, Brucellosis, MIC, Rifampin, Ceftriaxone, E-test, Egypt

## Abstract

**Background:**

Brucellosis poses a significant public health problem in Mediterranean countries, including Egypt. Treatment of this disease is often empirical due to limited information on the antibiotic susceptibility profiles of *Brucella* spp. in this region of the world. The aim of this study was to determine the antibiotic susceptibility profiles of *Brucella* blood isolates in Egypt, a country endemic for brucellosis.

**Methods:**

*Brucella* spp. isolates were identified from the blood cultures of acute febrile illness (AFI) patients presenting to a network of infectious disease hospitals from 1999–2007. Minimum inhibitory concentrations were determined for tetracycline, gentamicin, doxycycline, trimethoprim-sulfamethoxazole, streptomycin, ceftriaxone, ciprofloxacin and rifampin using the E-test. Interpretations were made according to Clinical and Laboratory Standards Institute (CLSI) guidelines.

**Results:**

A total of 355 *Brucella* spp. isolates were analyzed. All were susceptible to tetracycline, doxycycline, trimethoprim-sulfamethoxazole, streptomycin and ciprofloxacin; probable resistance to rifampin and ceftriaxone was observed among 277 (64%) and 7 (2%) of the isolates, respectively. Percentages of isolates showing probable resistance to rifampin were significantly lower before 2001 than in the following years (7% vs. >81%, p < 0.01).

**Conclusions:**

Despite the high burden of brucellosis in Egypt and frequent empirical treatment, isolates have remained susceptible to the majority of tested antibiotics. However, this is the first report of high rates of probable resistance to rifampin among *Brucella* isolates from Egypt. Patients should be closely monitored while following standard treatment regimens. Continued surveillance, drug susceptibility studies and updated CLSI interpretive criteria are needed to monitor and update antibiotic prescribing policies for brucellosis.

## Introduction

Brucellosis is endemic in many parts of the world, including Latin America, the Middle East, Africa, and Asia [1] and results in tremendous economic losses through reproductive failure in animals. Human disease is usually caused by *B. melitensis*[2,3] and is contracted mainly through exposure to *Brucella*-contaminated milk and contagious organs from infected animals [4,5]. In developing countries brucellosis is commonly present where small ruminants are kept. The disease was first reported in Egypt in 1939, and isolates characterized since then have belonged to *B. melitensis* biovar 3 [3]. In a previous surveillance study on acute febrile illness (AFI) patients in Egypt, the estimated annual incidence of brucellosis ranged from 64 to 70 per 100,000 population [6].

According to World Health Organization (WHO) guidelines, the recommended combination of treatment drugs for human brucellosis is doxycycline along with either rifampin or streptomycin [7], a recommendation that has been in place for more than a decade [8]. Although *Brucella* isolates are generally considered susceptible to these antibiotics, sporadic cases of antibiotic resistance and disease relapse have been reported [9,10]. However, routine antimicrobial susceptibility testing is generally not conducted for *Brucella* due to its fastidious growth requirements, risk of laboratory-acquired infections and need for biological safety level 2 or 3 precautions. The aim of this study was to take advantage of an unprecedented opportunity to test a repertoire of *Brucella* isolates (n = 355) collected from Egypt over an eight year period to define the most common species and evaluate the minimum inhibitory concentrations (MICs) of eight commonly used antibiotics. Trends of bacterial resistance will be evaluated during the study period.

## Materials and methods

*Brucella* isolates were obtained from blood culture aspecimens collected between 1999 and 2007 during sentinel site surveillance activities for AFI conducted in 13 infectious disease hospitals from four regions in Egypt: Alexandria, the Nile delta, Cairo and Upper Egypt. The protocol was approved by NAMRU-3 institutional review board and informed consents were available from all patients who enrolled into this study. Isolates were identified based on colony morphology, Gram staining, oxidase and catalase testing, production of urease, the requirement of CO_2_ for growth, H_2_S production, sensitivity to dyes (e.g. basic fuchsin and thionin) and seroagglutination [11]. Confirmatory speciation testing was done using PCR [12].

Susceptibility to eight antibiotics – tetracycline, gentamicin, doxycycline, trimethoprim-sulfamethoxazole, streptomycin, ceftriaxone, ciprofloxacin and rifampin – was determined using the E-test (AB Biodisk, Solana, Sweden), which is reliable, reproducible, easily performed and produces similar results to those of conventional methods for Brucella [8]. Briefly, Mueller-Hinton agar plates supplemented with 5% sheep blood were inoculated with bacterial suspensions calibrated to 0.5 McFarland standard turbidity and E-test strips were applied. The plates were placed in a 5% CO_2_ incubator for 48 hours and the resulting growth was examined to determine the MIC for streptomycin, tetracycline, doxycycline and trimethoprim-sulfamethoxazole according to CLSI guidelines for potential bacterial agents of bioterrorism [13]. Because breakpoints for *Brucella* against the other antibiotics tested have not been officially established, guidelines for slow-growing bacteria (*Haemophilus* spp.) were employed as has been done elsewhere [8]. Trends of bacterial resistance over the study period were evaluated. The reference strains *H. Influenza* ATCC 10211 and *S. pneumonia* ATCC 49619 were used as controls.

## Results

All *Brucella* isolates were identified as *B. melitensis* by biochemical testing and PCR and were shown to be susceptible to tetracycline (MIC_90_ = 0.19 μg/ml), trimethoprim-sulfamethoxazole (MIC_90_ = 0.19 μg/ml), doxycycline (MIC_90_ = 0.25 μg/ml) and streptomycin (MIC_90_ = 2 μg/ml). In addition, all strains were susceptible to ciprofloxacin (MIC_90_ = 0.38 μg/ml) according to the criteria for slow-growing bacteria (Table 1). However, the MIC values for rifampin ranged from 0.25-4.0 μg/ml, and according to CLSI breakpoints for slow-growing bacteria (*Haemophilus* spp.), reduced susceptibility (MIC 2-3 μg/ml) in 158 isolates (45%) and probable resistance (MICs ≥ 4 μg/ml) in 69 isolates (19%) were demonstrated. Only seven isolates (2%) demonstrated probable resistance to ceftriaxone (Table 2).

**Table 1:** **MIC ranges, MIC**_**50**_**and MIC**_**90**_**values of eight antibiotics against *Brucella* isolates**

**Antibiotics**	**Range (μg/ml)**	**MIC**_**50**_	**MIC**_**90**_	**CLSI Breakpoints for *Brucella* (μg/ml)**		
				**S**	**I**	**R**
Trimethoprim-Sulfamethoxazole	0.006-0.75	0.047	0.19	≤ 2/38	-	-
Tetracycline	0.023-0.75	0.125	0.19	≤ 1	-	-
Doxycycline	0.016-0.5	0.125	0.25	≤1	-	-
Ciprofloxacin	0.125-0.75	0.25	0.38	≤ 1^a^	-	-
Ceftriaxone	0.064-4	0.5	1	≤ 2 ^a^	-	-
Streptomycin	0.125-3	1.5	2	≤ 8	-	-
Gentamicin	0.094-3	0.5	1	Not defined		
Rifampin	0.25-6	**2**	**4**	≤ 1 ^a^	2 ^a^	≥ 4 ^a^

^a^ CLSI breakpoints for slow-growing bacteria (*Haemophilus* spp.).

**Table 2:** **Distribution of MICs values among *Brucella* isolates from AFI patients**

**MICs**	**Antibiotics/No. of isolates**							
	**TS**	**TC**	**DC**	**CI**	**GM**	**TX**	**SM**	**RI**
0.006	2							
0.008	8							
0.012	10							
0.016	26		1					
0.023	40	3	1					
0.032	48	7	5					
0.047	62	24	19					
0.064	37	66	49			3		
0.094	28	67	72		1	2		
0.125	44	100	108	2	7	6	4	
0.19	19	57	58	12	21	14	4	
0.25	11	23	29	32	49	30	10	4
0.38	13	6	10	22	47	69	10	2
0.5	3	1	3	9	81	98	21	10
0.75	4	1		2	77	77	41	24
1					47	41	61	37
1.5					18	8	80	51
2					5	6	89	79
3					2		35	79
4						1		69
8								
Total	355	355	355	79	355	355	355	355

TS = trimethoprim-sulfamethoxazole, TC = tetracycline, DC = doxycycline. CI = ciprofloxacin, GM = gentamicin, TX = ceftriaxone, SM = streptomycin, RI = rifampin.

Although the number of isolates collected from the same regions per year was variable, the MIC for rifampin showed an increase over time. For example, the percentage of isolates showing probable resistance to rifampin was significantly lower (7%) in 85 isolates from 1999 and 2000 than in 270 isolates (81%) from 2001–2009 (p < 0.01) (Table 3).

**Table 3:** **Percentages of *Brucella* isolates with reduced susceptibility to rifampin (MIC ≥2 μg/ml) from 1999–2007**

**Year**	**Number of isolates tested**	**Number of isolates with rifampin(MIC ≥2 μg/ml**	**%**
1999	19	1	5
2000	66	6	9
2001	63	41	65
2002	13	13	100
2003	10	10	100
2004	7	7	100
2005	20	20	100
2006	100	81	81
2007	57	46	81
**Total**	**355**	**225**	**63**

## Discussion

Transmission of *B. melitensis* from small ruminants to humans has become a significant problem in Middle Eastern and Mediterranean countries [2,14]. Although some *B. abortus* has been diagnosed in Egypt [5,14], all *Brucella* spp. isolates identified in this study were *B. melitensis,* probably associated with transmission from small ruminants [3].

Screening 355 isolates against a panel of eight antibiotics by the E-test method showed that trimethoprim-sulfamethoxazole had the lowest MIC values, in agreement with previous reports from Italy and Peru [2,15], but considerably lower than other previous studies carried out in Turkey [16,17]. Nevertheless, higher rates of trimethoprim-sulfamethoxazole resistance have been reported in other countries, including Mexico [18] and Saudi Arabia [19].

Tetracycline was active in vitro against all isolates, which is consistent with previous reports [8,20]. Similarly, the related doxycycline, which is a major component of most therapeutic regimens against *Brucella* infection, showed relatively low MICs, although lower values have been reported elsewhere [16,17].

While ciprofloxacin testing showed a significantly high MIC_90_ value, the effectiveness of this drug against *Brucella* infection remains controversial. Both higher and lower MICs have been reported previously for this drug [17,21,22].

All *Brucella* isolates were susceptible to streptomycin in agreement with previous studies from various countries [8,18]. For gentamicin, breakpoints have not been defined by CLSI, but the range of isolate MICs varied from 0.094-3 μg/ml, which is relatively higher than other values reported previously (0.064-1.5 μg/ml) [8].

Rifampin demonstrated the highest MIC_90_ value, with 45% of the isolates showing reduced susceptibility and 19% probable resistance, according to CLSI criteria for slow-growing bacteria (Table 2). To our knowledge, this is the first report of high rates of reduced susceptibility to rifampin among *Brucella* isolates from Egypt, suggesting the emergence of isolates with variable degrees of resistance to this drug (CLSI criteria for slow-growing bacteria). Only a few isolates (8-9%) with reduced susceptibility to rifampin have been described before in Turkey [16,23], Kuwait [24] and Mexico [18]. In another study conducted in Peru, only one *Brucella* isolate demonstrated reduced susceptibility to rifampin (1.0 μg/ml) [15].

Despite the high burden of human and animal brucellosis in Egypt and frequent empirical treatment, isolates have remained susceptible to the majority of test antibiotics. Whether or not the high rates of probable resistance to rifampin among *Brucella* isolates from Egypt had an impact on the treatment of patients is not known. One report from Egypt [9] observed relapse in 59.3% of the patients with osteoarticular brucellosis who were treated for 5 months or less with two drugs (rifampicin + doxycycline). By contrast relapse occurred in 7.9% of patients who were treated for more than 5 months. This report also noted that there was no relapse in patients who received 3 drugs (rifampicin + streptomycin + doxycycline) in combination, although treatment with streptomycin for long periods has been associated with ototoxic or nephrotoxic manifestations [25].

## Conclusions

Future antimicrobial surveillance studies are critical to monitor patients for relapse or treatment failure and developing specific assessment breakpoints for testing the susceptibility of *Brucella* strains to rifampin and other appropriate antibiotics in Egypt and other endemic parts of the world.

## Competing interests

The authors declare that they have no competing interests.

## Authors’ contribution

MA-M developed the concept, processed the samples and participated in writing the manuscript. BH, MOW, GP, and ED developed the concept, analyzed the results, wrote and reviewed the manuscript. BA-R and GR processed the samples and helped in analyzing the results and writing the manuscript. All authors read and approved the final manuscript.

## Copyright assignment statement

Authors are employees of the U.S. Government. This work was prepared as part of their official duties. Title 17 U.S.C. §105 provides that ‘Copyright protection under this title is not available for any work of the United States Government.’ Title 17 U.S.C. §101 defines a U.S. Government work as a work prepared by a military service member or employee of the U.S. Government as part of that person’s official duties.

## Disclaimer

The views expressed in this article are those of the authors and do not necessarily reflect the official policy or position of the U.S. Department of the Navy, the U.S. Department of Defense, the U.S. Government, or the Egyptian Ministry of Health and Population.

## Financial support

This work was supported by a grant from the U. S. Agency for International Development (USAID) and the U.S. Department of Defense Global Emerging Infections Surveillance and Response System (DoD-GEIS) (work unit number E022).
